# Characterization of neural mechanotransduction response in human traumatic brain injury organoid model

**DOI:** 10.1038/s41598-023-40431-y

**Published:** 2023-08-19

**Authors:** Susana M. Beltrán, Justin Bobo, Ahmed Habib, Chowdari V. Kodavali, Lincoln Edwards, Priyadarshini Mamindla, Rebecca E. Taylor, Philip R. LeDuc, Pascal O. Zinn

**Affiliations:** 1 Department of Mechanical Engineering, Carnegie Mellon University, Pittsburgh, 15213 PA USA; 2 Department of Neurosurgery, University of Pittsburgh Medical Center, Pittsburgh, 15213 PA USA; 3 Hillman Cancer Center, University of Pittsburgh Medical Center, Pittsburgh, 15232 PA USA; 4 Department of Radiology, University of Pittsburgh Medical Center, Pittsburgh, 15232 PA USA

**Keywords:** Brain injuries, Stem-cell biotechnology

## Abstract

The ability to model physiological systems through 3D neural in-vitro systems may enable new treatments for various diseases while lowering the need for challenging animal and human testing. Creating such an environment, and even more impactful, one that mimics human brain tissue under mechanical stimulation, would be extremely useful to study a range of human-specific biological processes and conditions related to brain trauma. One approach is to use human cerebral organoids (hCOs) in-vitro models. hCOs recreate key cytoarchitectural features of the human brain, distinguishing themselves from more traditional 2D cultures and organ-on-a-chip models, as well as in-vivo animal models. Here, we propose a novel approach to emulate mild and moderate traumatic brain injury (TBI) using hCOs that undergo strain rates indicative of TBI. We subjected the hCOs to mild (2 s^−1^) and moderate (14 s^−1^) loading conditions, examined the mechanotransduction response, and investigated downstream genomic effects and regulatory pathways. The revealed pathways of note were cell death and metabolic and biosynthetic pathways implicating genes such as CARD9, ENO1, and FOXP3, respectively. Additionally, we show a steeper ascent in calcium signaling as we imposed higher loading conditions on the organoids. The elucidation of neural response to mechanical stimulation in reliable human cerebral organoid models gives insights into a better understanding of TBI in humans.

## Introduction

Traumatic brain injury (TBI) hospitalized 223,135 people in 2019 and caused the death of 64,362 people in 2020 in the United States^1^. TBI is incurred to the brain due to a blow, foreign object penetrating the skull, or acceleration-deceleration forces^2^. Most injuries occur during collision sports, motor vehicle accidents, and military combat. There are mitigation strategies like wearing proper headgear and seat belts to reduce the risk of head injuries; nevertheless, people are still susceptible to TBI as these countermeasures do not completely inhibit the mechanical impact on the brain. Research shows traumatic brain injury is linked to symptoms like memory loss, headache, fatigue, and photosensitivity^3,4^. Additionally, TBI presents a risk factor for Alzheimer’s disease and neurodegenerative syndromes, including Chronic Traumatic Encephalitis (CTE)^5^. Mostly, TBI sequelae are not curable but can only be partially addressed and symptomatically treated with pharmaceutical-, physical-, cognitive-, speech-, psychological-, occupational-, and behavioral therapy^6–10^. Methods to quantify TBI-related changes in gene expression remain elusive, and there are no treatments to reverse any damage to the brain after injury.

The mechanical stimulation of cells plays a continuous role in the development, growth, remodeling, and maintenance of biological systems. The mechanotransduction response of cells can trigger a range of chronic diseases, including osteoarthritis^11,12^, atherosclerosis^13,14^, and pulmonary fibrosis^15,16^. Evidence shows that TBI can trigger similar inflammatory responses in the brain^17–19^. However, investigations of potential impact of TBI on gene expression, epigenetic modifications, and other processes that can influence the functioning of genes and cellular pathways over an extended period following the injury are nascent. Most studies are in mice and rats^20^ and human testing of TBI is primarily limited to postmortem studies or clinical imaging^21,22^. The population with the highest risk of TBI (e.g., athletes and military personnel) demonstrate similar clinical course and outcomes, including TBI-induced structural brain abnormalities and post-TBI psychiatric complications^23–28^. Thus, the high prevalence of TBI and TBI-induced clinical complications warrant the need to characterize the genomic landscape and develop a suitable in-vitro model for this disease.

Organoids produced from iPSCs (induced pluripotent stem cells) or hESCs (human embryonic stem cells), have been proven effective in modeling various diseases, including microcephaly, autism, schizophrenia, and cancer^29–33^. These approaches have provided tremendous benefits that complement mice models with many enhanced benefits. The most immediate benefit is that these organoids are composed of human tissue, likely having a better representation for a 3D arrangement for human disease models. Further, organoids provide an advantage in genomic editing, allowing for the creation of specific mutations to mimic specific diseases where the genomic insult is known. They can also introduce corrections to the genome to restore wild-type function where hCOs have been created directly from patients with specific diseases. Finally, hCOs allow us to model more complex diseases by introducing genetic mutations into these human cerebral models, understanding disease initiation and progression better, and making better treatment decisions that mouse models could not achieve^34,35^.

Ramirez et al. conducted a groundbreaking study that merits recognition, as they were the first to utilize cerebral organoids for modeling traumatic brain injury (TBI). Their innovative approach using penetrative techniques and slow compression methods on human cerebral organoids and mouse models provided valuable insights into TBI mechanisms^36^. Our work takes a different path by focusing on only human-specific models for TBI research. Similarly, Shoemaker et al. made significant contributions to the field by utilizing fluorescence and absorption techniques to visualize trauma-related differences in human cortical spheroids^37^. Their work shed light on certain aspects of TBI pathology. Our study employed RNA sequencing as a powerful tool to identify and analyze gene products associated with different TBI conditions.

Our work highlights the ability to capture and analyze gene expression patterns at different TBI stimulation profiles. While previous TBI hCO research observed TBI effects through fluorescence imaging, our approach enables a deeper understanding of the underlying molecular mechanisms involved. By leveraging different techniques and focusing on human-specific models, our research offers a distinct perspective that complements and expands upon their findings.

**Figure 1:**
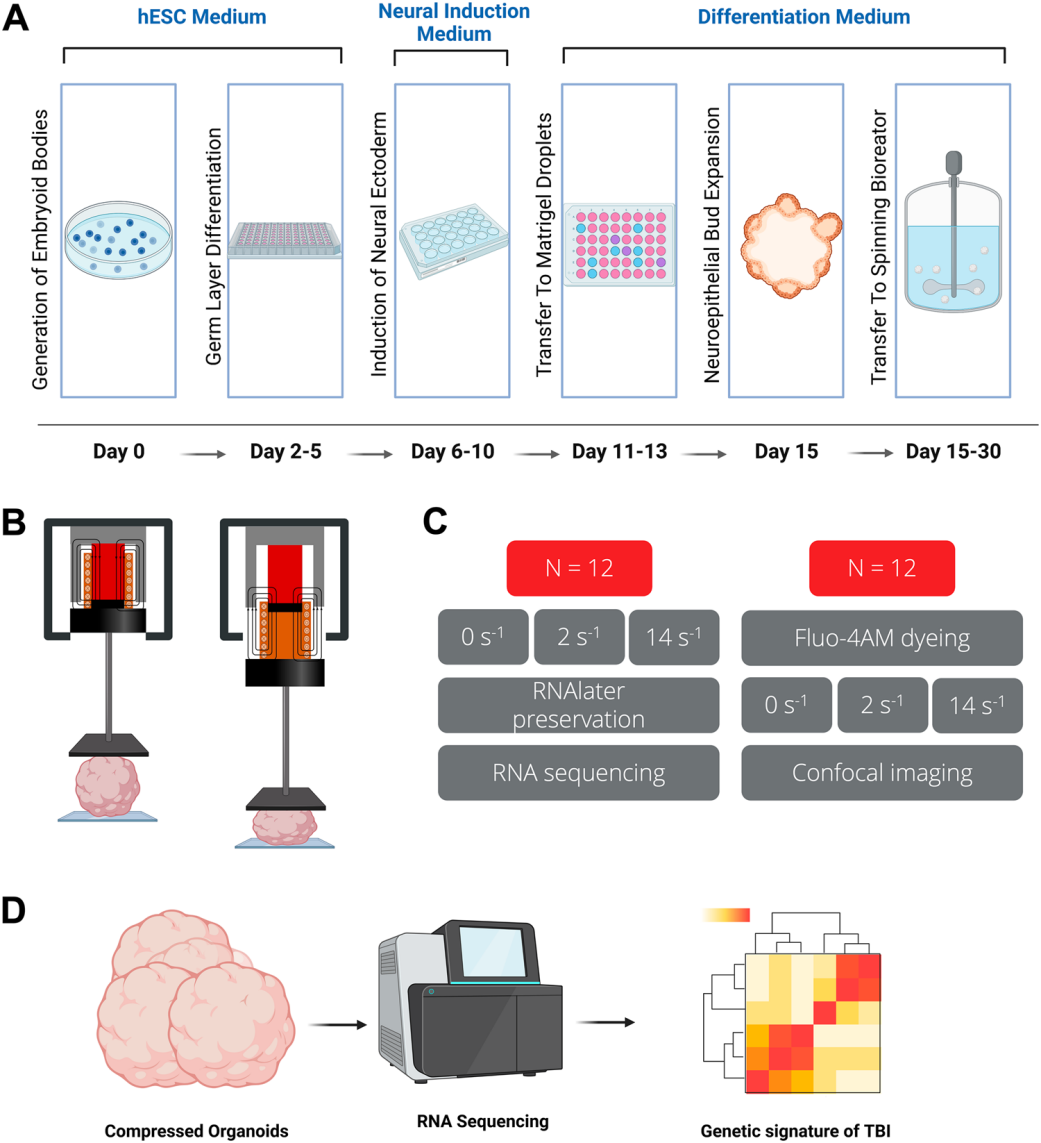
Mechanical stimulation of human cerebral organoids toward understanding human neural response after traumatic brain injury. (**A**) Human cerebral organoid (hCO) fabrication method to create the multicellular systems for mechanical stimulation. (**B**) Schematic of the mechanical excitation testbed (MET) system to uniaxially compress hCOs with the experimental procedures. (**C**) Process of experimentation (**D**) RNA sequencing on the compressed organoids was investigated to understand better the neural function implications of the mechanical stimulation. Illustrations were designed using Biorender.

We propose a novel approach to emulate mild and moderate TBI on human cerebral organoids by uniaxially compressing them with relevant strain rates and observing how TBI influences neuron signaling and gene expression in the human cerebral organoids (Fig. 1). We assessed the extent of hCO alteration by visualizing the gene expression changes across samples subjected to various TBI stimulation profiles through RNA sequencing. This method of modeling traumatic head injury and identifying its genetic product repercussions to the brain can be used to determine accurate, timely, and personalized treatments for TBI patients. This approach also may provide a more conclusive TBI diagnosis by analyzing the genetic expression signature of the neural cells after experiencing mechanically induced impact.

## Results

We developed an integrated approach to mechanically stimulate human cerebral organoids. Our method combines multi-cellular systems within a more neural mimetic approach to better understand the neural response to mechanical stimulation similar to TBI. We used our custom-designed mechanical stimulation system through a highly controlled voice coil actuator to stimulate the organoids, varying the strain rates to analyze different mechanical signatures related to TBI. In this, we collected the organoids and analyzed them using RNA sequencing to understand the genetic responses of our multi-cellular systems. Moreover, we examined real-time cellular calcium response to understand how cells responded to a known mechanotransduction calcium signaling pathway. This approach also allowed us to compare the genetic response signatures to other well-known neural and mechanically related findings, further enhancing our understanding of how mechanical loading affects neural-related systems.

### Organoid production

**Figure 2:**
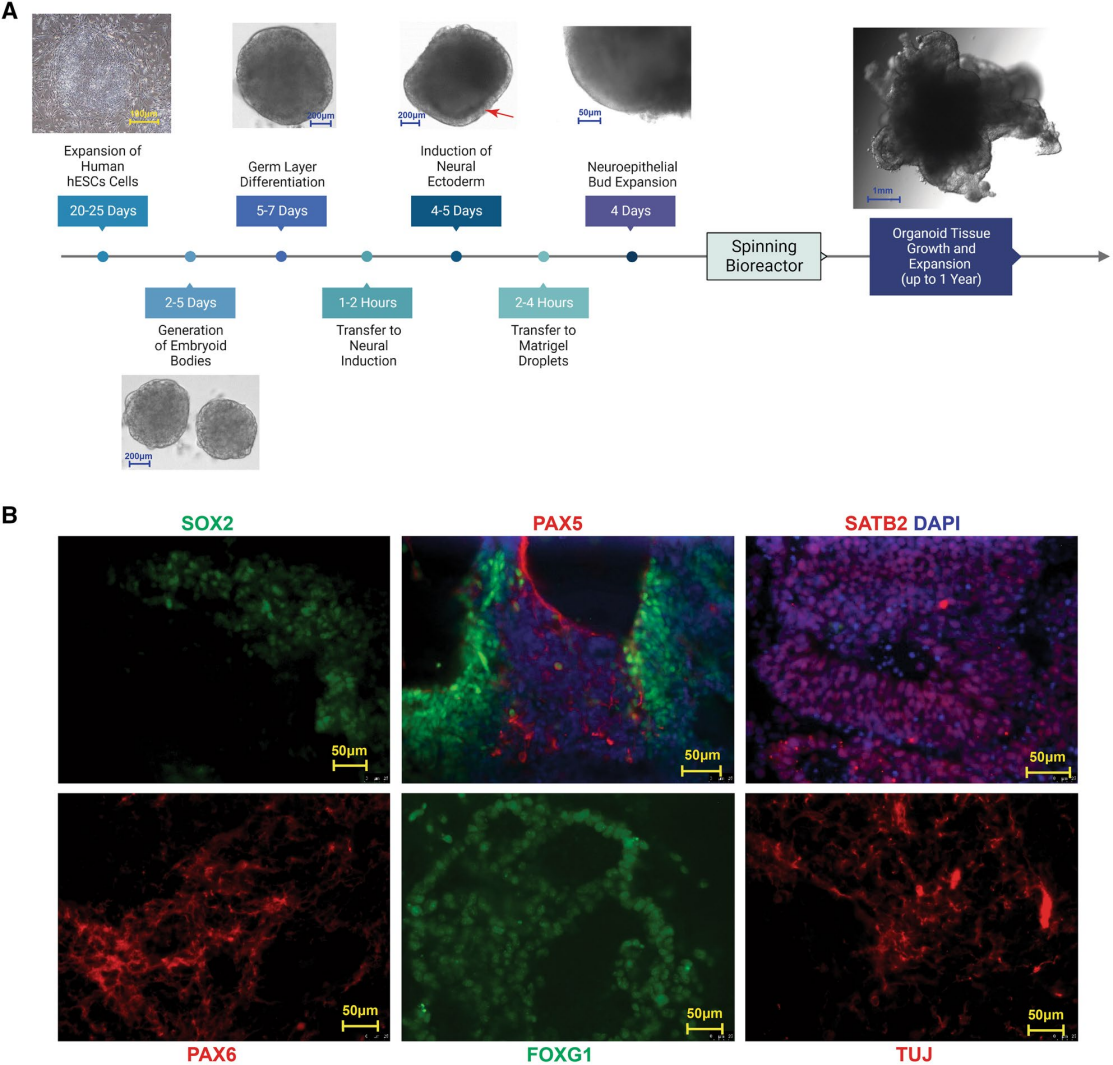
Organoid synthesis and assessment. (**A**) Timeline for the generation of the cerebral organoids. Representative images of the timeline starting with hESCs in culture followed by the sequential images of the key representative steps in the generation of cerebral organoid formation. The red arrow indicates induction of neural ectoderm. (**B**) Key developmental markers associated with cerebral organoids, 20$\times$ magnification. From left to right SOX2 (green), PAX6 (red), PAX5 (red), FOXG1 (green), SATB2 (red) and TUJ1 (red). DAPI is shown in purple.

**Figure 3:**
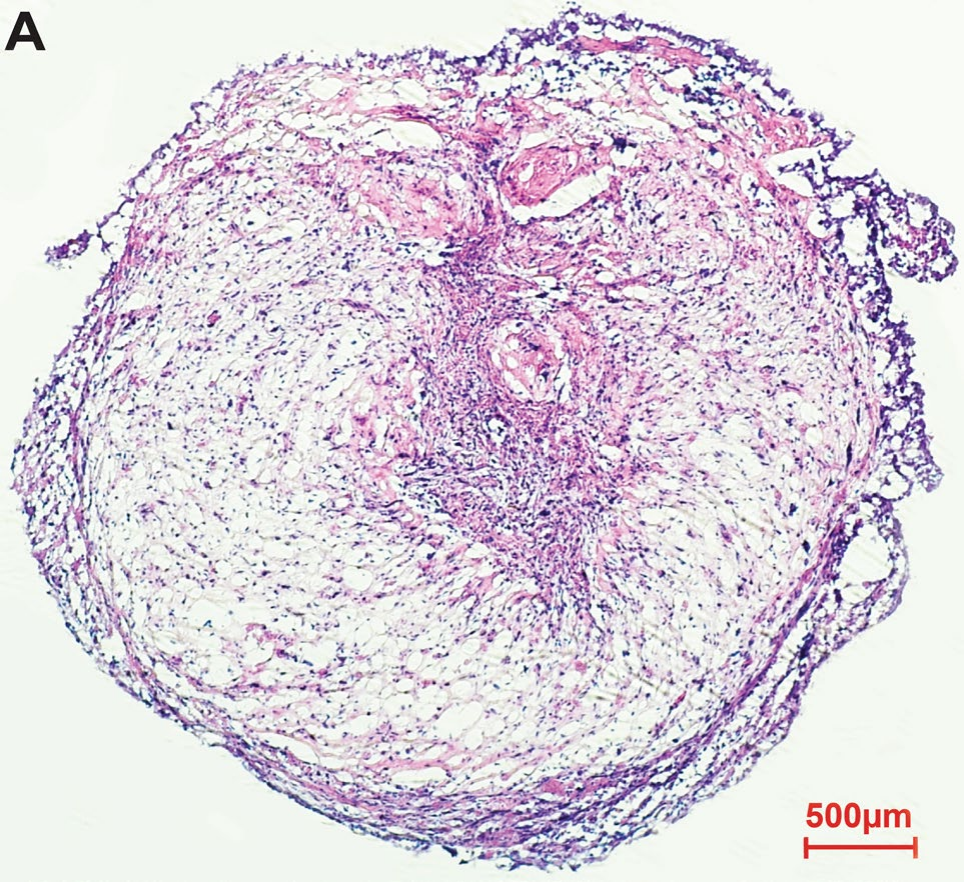
Hematoxylin and eosin staining of hCOs displaying typical normal morphology.

We formed optimum hCOs using a modified version of the Linkous protocol^33^. We achieved fully formed organoids to model the human brain starting with human embryonic stem cells, which we cultured in a 4-week period, to develop embryoid bodies, neural ectoderm, and cerebral organoid tissue (Fig. 2A). Consistent with cerebral organoids achieved by others^29,33^, we identified the expression of pluripotency markers such as SOX2 and prosencephalic differentiation markers such as PAX6, which revealed forebrain cellular identity (Fig. 2B). Further, we tested for brain regionalization by staining FOXG1, a cerebral cortex form and function marker (Fig. 2B). We also identified potential neurodevelopmental markers such as PAX5 and the post-natal neuronal marker SATB2 (Fig. 2B). TUJ staining indicated distinct neuronal identity (Fig. 2B). These markers suggest that our hCOs recapitulate cortical organization and cell types with several brain region identities present. These markers were chosen for this very reason, as they can confirm distinct boundaries between regions of the brain and identify cell types. For example, PAX6 defines the forebrain^38^. Further sub-regionalization within the established forebrain results in the expression of cortical neuronal markers associated with defining the prefrontal cortex, such as FOXG1^38^. PAX5 is a neurodevelopmental marker that, in early brain development, defines the mid-hindbrain boundary^38^; however, it should be noted that over time and into adulthood, this marker is widespread throughout the brain^38^. SATB2 determines neuronal identity defining pyramidal neurons, specifically upper-layer neurons^39^. TUJ1 can also define a proliferative population within the development of the telencephalon^40^. Collectively, these data suggest our hCOs are consistent with aspects of the human cytoarchitecture of the brain. To assess whether hCOs resemble cerebral organoids and correspondingly normal brains, we looked at the histology of hCOs which showed standard cellular morphology characteristics of normal tissue by hematoxylin and eosin staining (Fig. 3).

**Figure 4:**
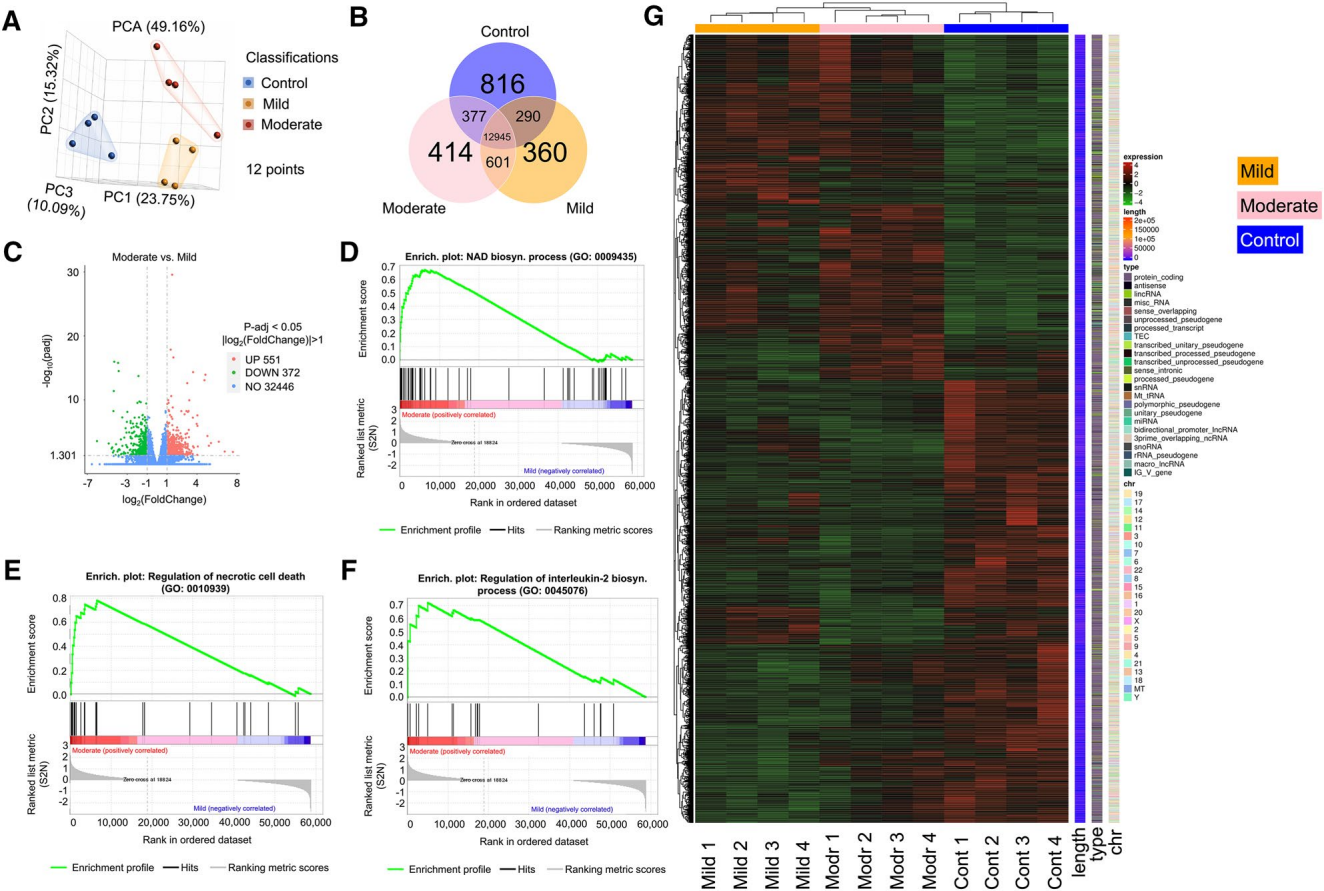
Genetic response after mechanical stimulation. (**A**) PCA analysis to emphasize the separation of groups of TBI of the hCOs we tested. (**B**) The Venn diagram represents the number of genes that are uniquely expressed within each group, with the overlapping regions showing the number of genes that are co-expressed in two or more groups. (**C**) The moderate vs. mild volcano plot shows the distribution of differently expressed genes. The x-axis is the fold change in the gene expression between different samples and the y- axis is the statistical significance of the differences. The most up-regulated genes are towards the right (red), and the most down-regulated genes are towards the left (green). The most statistically significant genes are towards the top. (**D**) Gene set enrichment plot indicating that NAD biosynthesis genes are enriched from the differentially expressed genes in the volcano plot. (**E**) Gene set enrichment plot indicating that necrotic cell death genes are enriched from the differentially expressed genes in the volcano plot. (**F**) Gene set enrichment plot indicating that Interleukin 2 regulatory genes are enriched from the differentially expressed genes in the volcano plot. (**G**) Unsupervised cluster analysis of differently expressed genes. There are 4 samples in each group. Different colors indicate differential expression (red, up-regulation; green, down-regulation; black, no change in expression). Informatics visualizations were made using Partek Inc. (2020). Partek Flow (Version 10.0). https://www.partek.com/partek-flow/.

### RNA sequencing after injury

In order to gain more insight into gene expression in these mechanically stimulated organoids, we ran RNA sequencing on 12 hCOs: four control, four mildly, and four moderately stimulated hCOs. The control hCOs did not undergo mechanical stimulation. We used a principle component analysis (PCA) approach^41^, which revealed separation between all three groups of hCOs (Fig. 4A). There were over 12,000 genes in common between all three groups of hCOs, whereas 601 genes were common to both mild and moderate conditions of mechanotransduction in hCOs (Fig. 4B). We focused on the mild versus moderate mechanotransduction condition to find gene pathways that were significant in this group (Fig. 4C). Using gene set enrichment, we determined three major pathways: an NAD biosynthesis pathway (Fig. 4D), a necrotic cell death pathway (Fig. 4E), and a regulatory pathway of interleukin 2 (Fig. 4F). We have identified additional pathways with features and functions apart from the main pathways indicated. One pathway comes from the mild TBI cohort (Chromatin remodeling) and the other from the moderate TBI cohort (INO80 complex), which is an ATP-dependent chromatin remodeler suggesting that at the mild and moderate levels, chromatin remodeling occurs. This is important given the various functions chromatin remodeling can perform including but not limited to gene expression and DNA repair. We do not have further details into the mechanisms involved with these pathways other than to indicate according to the gene set enrichment data see Supplemental Fig. 1, that chromatin remodeling is being positively regulated.

The necrotic cell death pathway is characterized as a non-apoptotic pathway that triggers cell death; instead, cell death is triggered by an overwhelming chemical or physical insult with features of cell swelling or rupture prior to cell death^42–44^ and found in trauma^45^. Interestingly, calcium is a significant factor in the execution of this process^46^. The NAD biosynthesis pathway acts as a critical regulator of physiologic homeostasis in the face of infection, circadian disorder, and inflammation^47^. The interleukin 2 (IL-2) pathway has had increasing evidence of its involvement in trauma. For example, there is evidence of high levels of IL-2 receptor in trauma^48^; however the trauma was burn patients. More specifically, in TBI patients, IL-2 has been shown to activate regulatory T cells to suppress neuronal inflammation and, therefore, may act as a means of trying to alleviate the symptoms associated with trauma^49^. To explore the particular genes that are associated with mild and moderate mechanotransduction of hCOs, we examined the differential expression of hCOs under mild and moderate mechanotransduction. We ran an unsupervised analysis of all three conditions of hCOs that were studied and visualized in a heatmap where the gene expression for control, mild and moderate hCOs show distinct gene activation (Fig. 4G). Taken together, these data suggest genetic expression differences across samples subjected to various TBI stimulation profiles.

**Figure 5:**
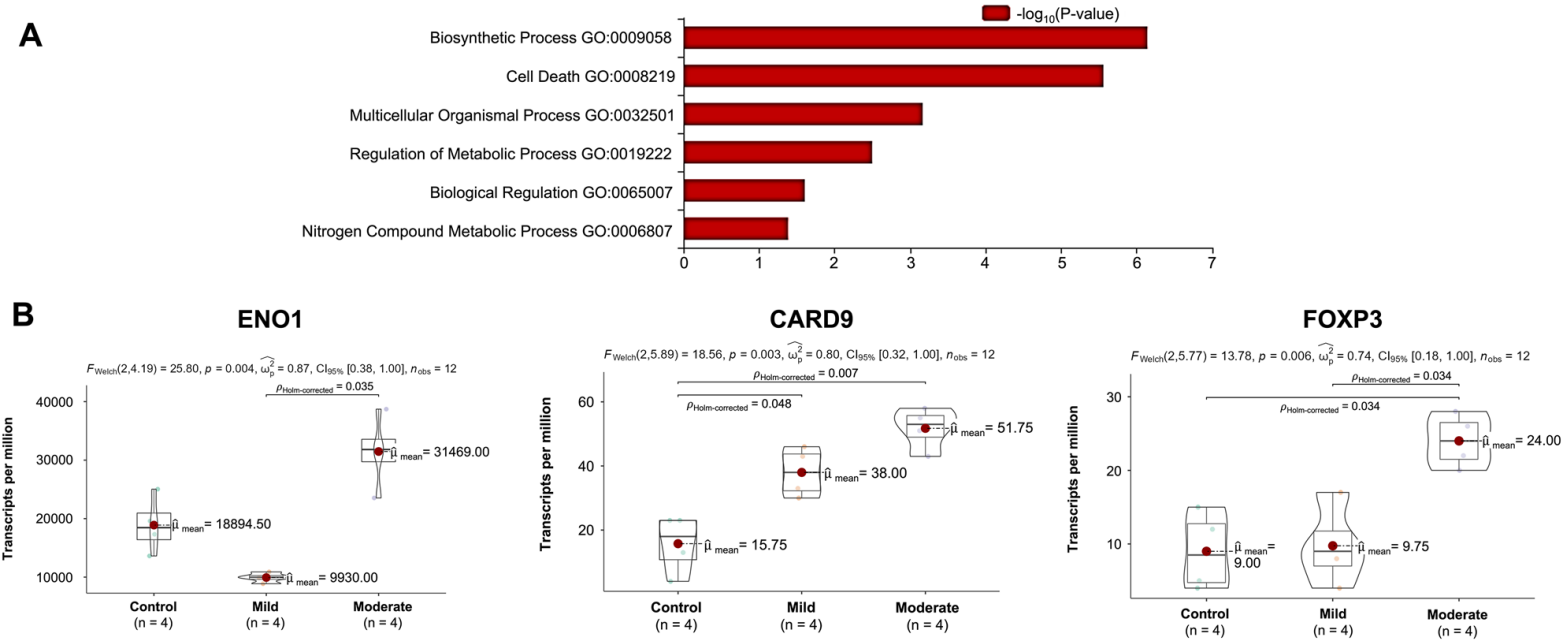
Significant functional genes under TBI mimetic stimulation. (**A**) GO enrichment analysis histogram where the 6 most significant genetic functions present in the TBI samples are displayed. The x-axis is the significance level of GO Term enrichment. The y-axis is the genetic function (GO Term). (**B**) Violin plots depicting the transcript levels of ENO1, CARD9, and FOXP3 for each TBI condition. Informatics visualizations were made using Partek Inc. (2020). Partek Flow (Version 10.0). https://www.partek.com/partek-flow/.

Building on our analysis from RNA sequencing, we explored the gene ontology as it related to hCO mechanotransduction (Fig. 5A). Consistent with our geneset enrichment analysis, we observed biosynthesis processes and cell death as the top gene ontology processes (Fig. 5A). At the transcriptional level we confirmed, by our differential expression of mild versus moderate conditions, three genes were elevated: ENO1, CARD9, and FOXP3 (Fig. 5B). ENO1 (Enolase 1) has been associated with diseases such as Hashimoto’s Encephalopathy^50^. ENO1’s primary functions include RNA binding and transcription corepressor activity. CARD9 (Caspase Recruitment Domain Family Member 9) promotes cell death, and its activation has been associated with inflammation^51^. FOXP3 (Forkhead Box P3) has been implicated in Immunodysregulation^52,53^ with its primary functions include DNA-binding transcription factor activity and sequence-specific DNA binding.

**Figure 6:**
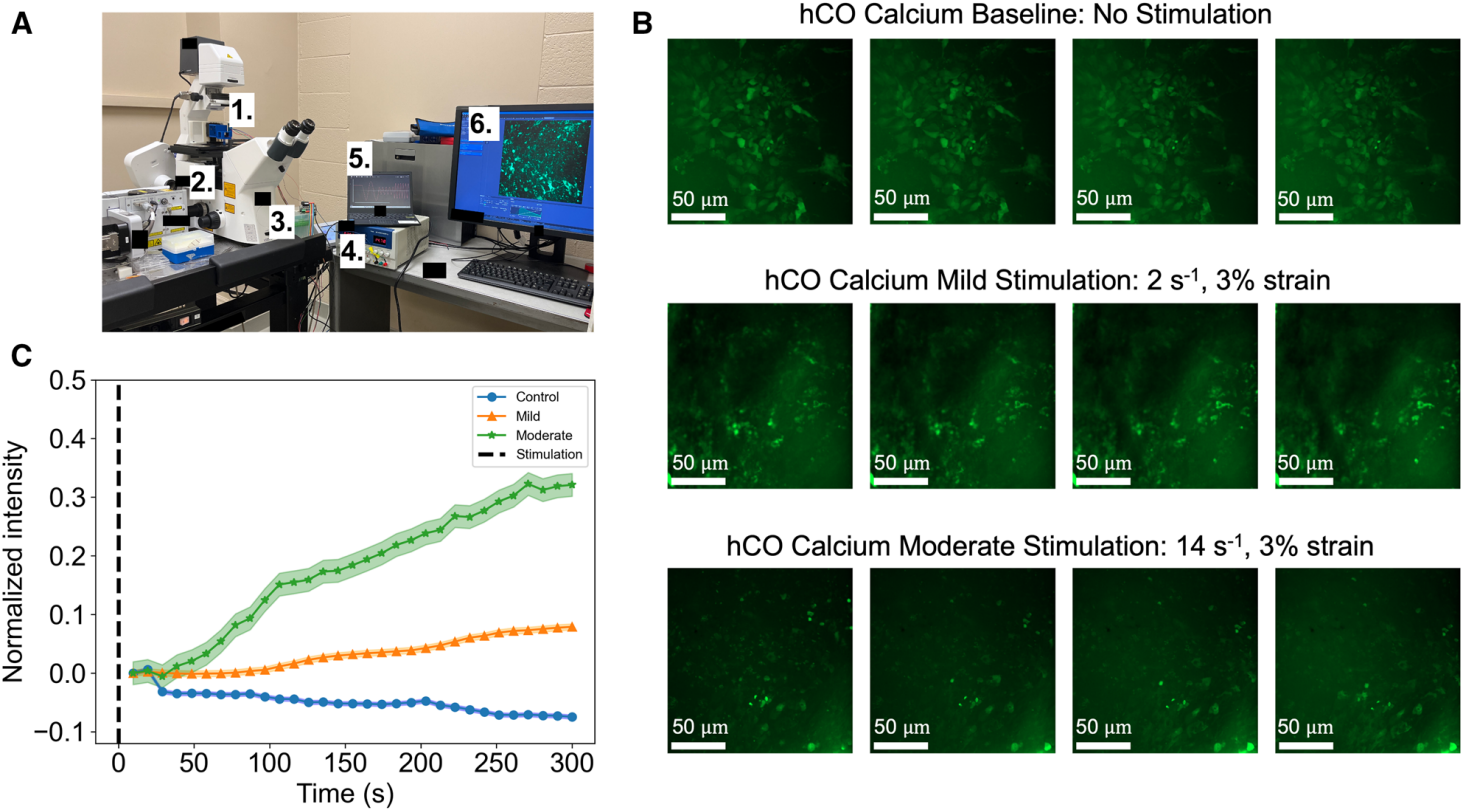
Mechanical stimulation of organoids and calcium response. (**A**) The MET approach for hCOs compression allowed for TBI comparisons to our selected strain rates for control, mild, and moderate stimulation. 1. Mechanical excitation testbed (MET). 2. Zeiss spinning disk confocal microscope. 3. Microcontroller for voice coil actuator. 4. Power supply. 5. Software to program microcontroller and oscilloscope. 6. Imaging computer. (**B**) Calcium signaling images of the uncompressed and compressed hCOs. (**C**) Graph of calcium signaling indicating increasing calcium signaling as the strain rate increases. n = 31 for each curve; shaded area represents standard error.

### Calcium signaling after injury

To mimic TBI injury on human cerebral organoids (hCOs) and view calcium signaling after injury, we used our Mechanical Excitation Testbed (Fig. 6A). The TBIs we induced on our hCOs are relevant to the mild and moderate characteristics of head injury in humans (Fig. 6B). We viewed the calcium signaling responses in the organoids after inflicting injury with relevant strain rates to understand the fast response mechanisms that are known to occur in many cells (Fig. 6C). In our cellular calcium response investigation, we showed the control condition had a mean Ca^2+^ intensity gradient of $-1.97 \times 10^{-4}$ with an R^2^ value of 0.832 over 5 min, with the lowest mean Ca^2+^ intensity values out of the three groups. The mild condition of 2 s^−1^ at 3% strain had a mean Ca^2+^ intensity gradient of $3.15 \times 10^{-4}$ with an R^2^ value of 0.962 over 5 min. The moderate condition of 14 s^−1^ at 3% strain had a mean Ca^2+^ intensity gradient of $1.22 \times 10^{-3}$ with an R^2^ value of 0.977 over 5 min, with the highest Ca^2+^ intensity values out of the three groups. We demonstrated that as the loading strain rate increased for each sample, the mean Ca^2+^ intensity also increased over time. There is an increased signaling response in neuronal cells with increased injury severity, thus, giving us an observation of crucial mechanotransduction responses that can affect neuronal health.

## Discussion

Our research focuses on using human cerebral organoids, which are human-specific 3D brain models, to address significant challenges in treating traumatic brain injury. Current-day treatment for TBI includes rest, over-the-counter pain relievers, anti-seizure drugs, diuretics, coma-inducing drugs, and surgery—all depending on the degree of brain injury. After these initial treatments, rehabilitation is the next step for treatment. Some patients improve with these methods, and others do not. This treatment method is the extent of clinical practice.

The physical consequences of traumatic brain injury are more readily apparent than nonphysical ones, which motivated our research to investigate the extent of gene expression changes following such injuries. Our study has revealed previously concealed genetic expression effects that can impact gene functioning and cellular pathways, as evidenced by our genetic and cellular mechanical stimulation experiments. Specifically, using our human-specific model, we conducted genetic analyses that demonstrated specific genetic products based on the severity of the injury.

Furthermore, our investigations demonstrated an increasingly pronounced calcium signaling response in neuronal cells as we applied greater mechanical stimulation, indicating a heightened mechanotransduction reaction under escalating mechanical insults. Utilizing human cerebral organoids (hCOs) provided a physiologically relevant model specific to humans, enabling us to gain valuable insights into the neuronal response to traumatic brain injuries.

Notably, the genes expressed under increasing mechanical insult have been associated with neurodegenerative diseases like Hashimoto’s Encephalopathy, genes that regulate cell death and inflammation, and immune dysregulation, all of which can negatively affect patients with TBI. These findings highlight the relevance and potential clinical implications of our research.

Through our work, we identified genetic products that arise from TBI. This critical step enables us to pave the way for personalized treatments that focus on the specific patient and can precisely target essential gene products and biological pathways, ultimately halting or potentially reversing the progression of the TBI-induced disease. By leveraging drugs already in production, our research holds promise for developing effective therapies that can address the newly identified genetic products derived from TBI.

Previous research on traumatic brain injury (TBI) These studies employed various methodologies, including animal models^54–56^, postmortem examinations of human subjects^56–58^, and studies involving living human subjects^59,60^. Some previous research has explored the versatility of disease modeling using organoids to model Microcephaly^29^ and the Zika virus^61^. Additionally, TBI has been studied for TBI^36,37^. These studies have provided valuable insights into TBI’s phenotypic cellular effects, with one particular TBI study utilizing mouse models in conjunction with the organoid models. However, the previous studies on TBI did not investigate the differences in calcium or genetic products between mild and moderate traumatic brain injuries.

Human cerebral organoids present a remarkable opportunity to bridge the gap between studies conducted on animal models and direct human experimentation by providing a personalized, rapid, and robust laboratory-constructed model. By leveraging this innovative approach, we aim to advance the understanding of traumatic brain injury (TBI) and its disease onset.

## Methods

### Organoid synthesis and culture

We cultured human embryonic stem cells (hESCs) using a modified version of the Linkous protocol^62^. Briefly, we plated hESCs reaching 80% confluency in low attachment 96-well plates. From this, embryoid bodies formed. We fed them every other day for 5 days with a 50 $\upmu$M Rho-associated protein kinase (ROCK) inhibitor for the first 2 days. Next, we transferred the embryoid bodies to Matrigel-coated (Corning #354277) 6-well plates and changed the medium daily (STEMdiff Neural Induction, STEMCELL Technologies) for 6 days which completed neural rosette cluster formation. Then, we used Matrigel droplets to solidify the clusters at 37 °C. Next, we grew them without agitation for 48 h in a differentiation medium containing a 1:1 mixture of DMEM/F12 and Neurobasal, 1:200 N2 supplement (Thermo Fisher Scientific), 1:100 B27 supplement without vitamin A (Thermo Fisher Scientific), 87.5 $\upmu$L of 1:100 2-mercaptoethanol, 1:4000 insulin (Sigma), 1:100 Glutamz (Thermo Fisher Scientific) and 1:200 MEM-NEAA. Finally, we transferred the formed tissue to an orbital shaker containing a differentiation medium without vitamin A (Thermo Fisher Scientific) (Fig. 2A).

### The mechanical excitation testbed system

For the compression of organoids, we used the Mechanical Excitation Testbed (MET). The MET comprises a voice coil actuator, a z-axis positioner, and a precision end effector, giving us access to a wide range of frequencies to simulate TBI and precisely compress the organoid with controlled strain rates and strain for a controlled number of pulses. This system allows us to recreate a more realistic 3D simulation environment for the hCOs. Additional details on the MET can be found in Bobo 2020 (Fig. 1B)^63^.

### Strain and strain rate selection

We sought to mimic mild and moderate traumatic brain injury to model TBI on the cerebral organoids effectively (Fig. 1C). TBI in humans is categorized according to specific clinical characteristics that signify brain damage: abnormal brain images, loss of consciousness, alteration of consciousness, and amnesia, according to the Glasgow Coma Scale (GCS). The GCS is the most widely used scale to determine head injury severity^64^. However, since we are using cerebral organoids, we want to understand the mechanical conditions to mimic these injuries. We control the strain imposed on the organoid and the strain rate with the VCA and microcontroller, and we use these to mimic the mechanics of the brain under dynamic loading (Fig. 4). We applied a 3% strain on all organoids, excluding the control condition organoids, with differing strain rates of 2 s^−1^ for mild TBI and 14 s^−1^ for moderate TBI based on previous literature on mechanical loading using strain rates related to TBI in cells and brain tissue mechanics^65–68^. We chose a 3% strain because, at this strain, we would avoid crushing the 1 mm organoids and were still able to achieve the necessary strain rates for testing. Our goal was not to completely crush the organoids, but rather to induce a controlled mechanical perturbation that simulates the impact forces experienced during TBI. With our MET compressing the 1 mm hCOs at a 3% strain, we made several simplifying assumptions of the mechanical behavior of the hCOs. A 3% strain is considered a small deformation, so linear elasticity assumptions were used. This assumption implied that the deformation is small enough to neglect geometric nonlinearity effects and maintain a linear relationship between stress and strain. Due to the spherical shape of the hCOs, we also assumed that the deformation and strain distribution are spherically symmetric which allows for a simplified analysis by considering only radial displacements and strains.

### Compression of organoids for RNA sequencing analysis

After the organoids are grown, we uniaxially compressed them at the relevant TBI strain rates. We compressed one organoid at a time. We implemented testing of 12 organoids with three grouping of testing criteria: no strain rate applied, mild strain rate applied, and moderate strain rate applied; the no strain rate grouping was our control group. For each control case organoid, we removed it from the culture media and placed it on a $22 \times 40$ mm rectangular cover glass. Next, we aspirated the excess media while carefully avoiding the organoid on the coverslip. We used 250 $\upmu$L of RNAlater (Millipore Sigma Aldrich, R0901-500 mL), an aqueous, non-toxic tissue storage reagent, to stabilize and preserve cellular RNA for the cerebral organoids after compression and prior to freezing for later processing (Fig. 1D). For each mild case organoid, we removed it from the culture media, placed it on a $22 \times 40$ mm rectangular cover glass, and carefully aspirated the excess media while avoiding the organoid. We then placed the organoid on the microscope stage underneath the MET and lined up the organoid with the mechanical stimulation plunger. Next, we lowered the plunger to the surface of the organoid and compressed the organoid at a mild TBI strain rate of 2 s^−1^ at a 3% strain. The initial position for this motion was measured with a linear scale encoder which was readout on an oscilloscope. We used 250 $\upmu$L of RNAlater to stabilize and protect cellular RNA to later freeze the organoids for processing. For each moderate case organoid, we followed the same steps as the mild case, except we compressed the organoid at a moderate TBI strain rate of 14 s^−1^ at a 3% strain. After testing, we stored all the organoids at $-20\, ^{\circ }$C and later transferred the samples to $-80\, ^{\circ }$C.

### Compression of organoids for calcium analysis

To better understand neural identity, activity, and 3D organization of cerebral organoids as a response to mechanics through calcium release^69,70^, we introduced Fluo-4 AM (stock: 50 g, molecular weight: 1097 g/mol), which is a cell-permeant Ca^2+^ indicator, to the organoids prior mechanical experimentation. We used DMSO as a solvent to yield a concentration of 25 $\upmu$M of Fluo-4 AM. In a new 24-well plate, we added 500 $\upmu$L of the 25 $\upmu$M dye into four wells. We placed three organoids in each well. Then, we incubated the hCOs (37 °C, 5% CO$_{2}$) for 45 min before we imaged them. After incubation, we removed a cerebral organoid from the Fluo-4 AM containing medium and placed it on a $22 \times 40$ mm rectangular cover glass while we aspirated excess media away. We placed the cover glass holding the Fluo-4 AM stained organoid on the Zeiss Spinning Disk Confocal Microscope (20$\times$ objective, 488 nm excitation wavelength, 514 nm emission wavelength). The compression conditions for the organoids were the same as the Compression of Organoids for RNA Sequencing Analysis: no strain rate applied, mild strain rate applied (2 s^−1^) at a 3% strain, and moderate strain rate applied (14 s^−1^) at a 3% strain (Fig. 4B).

### RNA sequencing analysis

We did RNA sequencing on the organoids, which included differential gene expression analysis and functional analysis to observe how TBI influenced the genetic expression in the cerebral organoids. After obtaining the raw data, we obtained an error rate distribution, GC-content distribution, and Data Filtering for a quality check. Next, we examined a mapping of the genes to the brain reference genome. Then, we split between the gene expression quantification, where we received correlation analysis-quality contract check on samples between samples. Finally, we implemented differential expression analysis and functional analysis focusing on enrichment analysis in Gene Ontology.

### Calcium analysis

To observe neuron signaling in the human cerebral organoids, we measured the intensity of the Fluo-4 AM dyed hCOs with a Zeiss Spinning Disk Confocal Microscope (Axio Observer Z1 System, $20\times$ objective, 488 nm excitation wavelength, 514 nm emission wavelength) at room temperature. We took an image every 10 s for 5 min for each control condition organoid at a 3 s exposure. For each mild and moderate condition organoid, we began imaging right after stimulation and took an image every 10 s for 5 min at a 3 s exposure. We measured the average gray value for each 8-bit image using Fiji^71^, which takes the sum of the gray values of all the pixels in our 512 $\times$ 512-pixel image and divides it by the number of pixels. The average gray values were exported and analyzed with a custom Python script.

### Supplementary Information

Supplementary Information.

## Data Availability

The datasets generated and analysed during the current study are available in the NIH repository, https://dataview.ncbi.nlm.nih.gov/object/PRJNA944161?reviewer=qt2d15phuuiej61foarfkciqgt.
