# Author Correction: Characterization of neural mechanotransduction response in human traumatic brain injury organoid model

**DOI:** 10.1038/s41598-024-52553-y

**Published:** 2024-01-25

**Authors:** Susana M. Beltrán, Justin Bobo, Ahmed Habib, Chowdari V. Kodavali, Lincoln Edwards, Priyadarshini Mamindla, Rebecca E. Taylor, Philip R. LeDuc, Pascal O. Zinn

**Affiliations:** 1 Department of Mechanical Engineering, Carnegie Mellon University, Pittsburgh, 15213 PA USA; 2 Department of Neurosurgery, University of Pittsburgh Medical Center, Pittsburgh, 15213 PA USA; 3 Hillman Cancer Center, University of Pittsburgh Medical Center, Pittsburgh, 15232 PA USA; 4 Department of Radiology, University of Pittsburgh Medical Center, Pittsburgh, 15232 PA USA

Correction to: *Scientific Reports* 10.1038/s41598-023-40431-y, published online 19 August 2023

In the original version of this Article, Philip R. LeDuc was omitted as a corresponding author. Correspondence and requests for materials should also be addressed to prl@andrew.cmu.edu.

The original Article has been corrected.

