# Supplementary material for: Characterization of neural mechanotransduction response in human traumatic brain injury organoid model
Source: Sci Rep. 2023 Aug 19;13:13536. doi: 10.1038/s41598-023-40431-y (PMC10439953; doi:10.1038/s41598-023-40431-y)
Supplement: Supplementary file 1 — Supplementary Information. [file 41598_2023_40431_MOESM1_ESM.pdf]

Supplemental information

# Characterization of Neural Mechanotransduction Response in Human Traumatic Brain Injury Organoid Model

**Susana M. Beltrán, PhD[1], Justin Bobo, PhD[1], Ahmed Habib, MD, MS.C [2,3], Chowdari V. Kodavali, PhD[2,3], Lincoln Edwards, PhD[2,3], Priyadarshini Mamindla, MS[4], Rebecca E. Taylor, PhD[1], Philip R. LeDuc, PhD[1], and Pascal O. Zinn, MD, PhD[2,3],***

1Carnegie Mellon University, Department of Mechanical Engineering, Pittsburgh, PA, 15213, USA

2University of Pittsburgh Medical Center, Department of Neurosurgery, Pittsburgh, PA, 15213, USA

3University of Pittsburgh Medical Center, Hillman Cancer Center, Pittsburgh, PA, 15232, USA

4University of Pittsburgh Medical Center, Department of Radiology, Pittsburgh, PA, 15232, USA

* zinnpo@upmc.edu

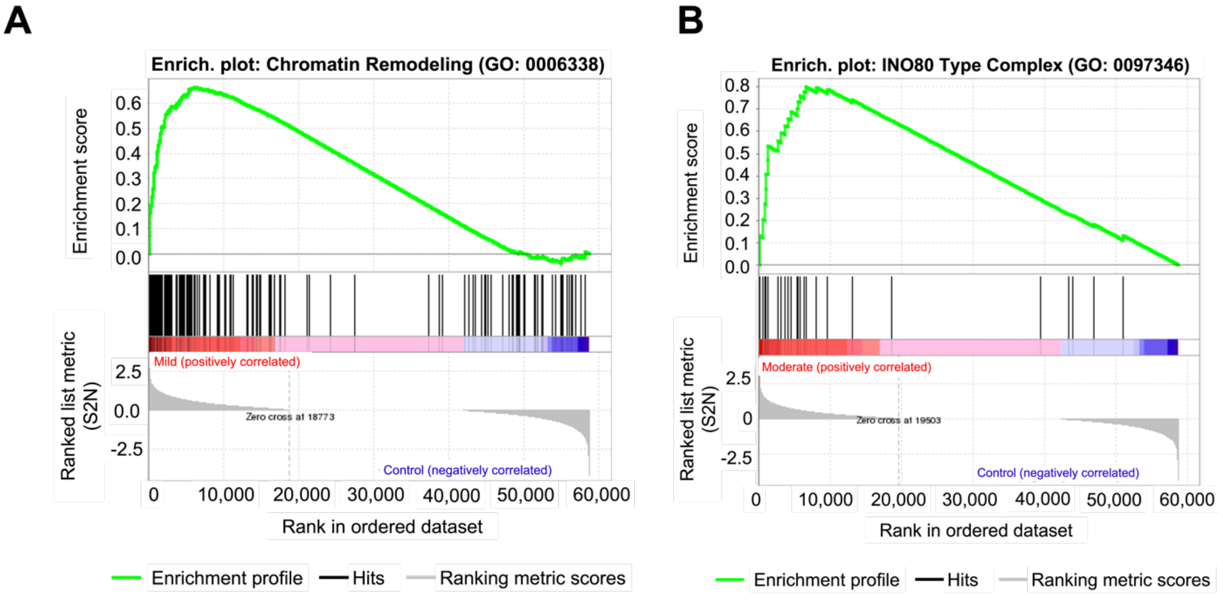


**Supplemental Figure 1:** Additional pathways with features and functions apart from the main pathways indicated: Chromatin remodeling is positively regulated in the Mild condition, INO80 Complex is positively regulated in the Moderate condition.

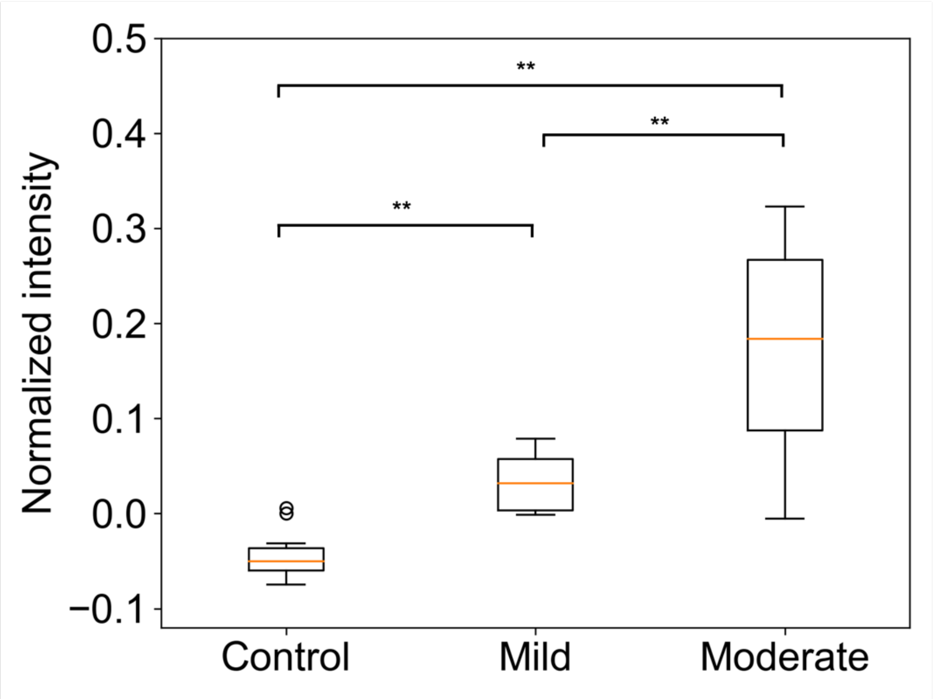


**Supplemental Figure 2:** Significance between groups in calcium imaging. n = 31 for each group. ** when $p < 0.01$ using the Mann-Whitney U statistical test.

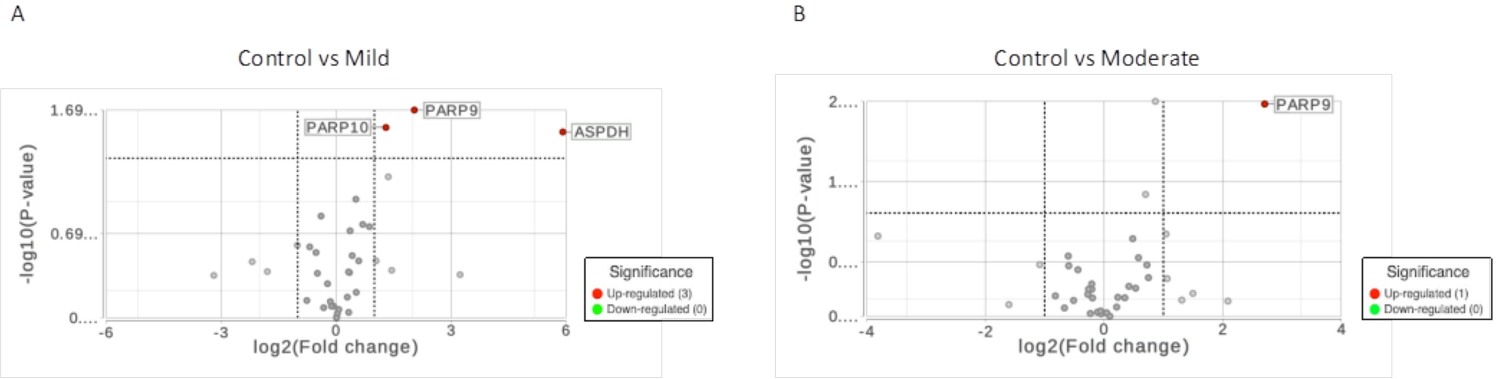


**Supplemental Figure 3:** A) The volcano plot between Control vs Mild samples showing the differential gene expression of PARP10, PARP9, and ASPDH genes which are part of the gene set enrichment representing the NAD biosynthetic process (GO:0009435). B) The volcano plot between the Control vs Moderate samples showing the differential gene expression of PARP9 gene which is part of the gene set enrichment representing the NAD biosynthetic process (GO:0009435). The x-axis is the fold change in the gene expression between different samples and the y- axis is the statistical significance of the differences. The most up-regulated genes are towards the right (red), and the most down-regulated genes are towards the left (green)
